# The Effectiveness of Artificial Intelligence in Detection of Oral Cancer

**DOI:** 10.1016/j.identj.2022.03.001

**Published:** 2022-05-14

**Authors:** Natheer Al-Rawi, Afrah Sultan, Batool Rajai, Haneen Shuaeeb, Mariam Alnajjar, Maryam Alketbi, Yara Mohammad, Shishir Ram Shetty, Mubarak Ahmed Mashrah

**Affiliations:** a Department of Oral and Craniofacial Health Sciences, College of Dental Medicine, University of Sharjah, United Arab Emirates; b Department of Dental Implantology, Guangzhou Medical University, China

**Keywords:** Oral cancer, Artificial intelligence, Neural network, Machine learning, Diagnosis

## Abstract

**Aim:**

The early detection of oral cancer (OC) at the earliest stage significantly increases survival rates. Recently, there has been an increasing interest in the use of artificial intelligence (AI) technologies in diagnostic medicine. This study aimed to critically analyse the available evidence concerning the utility of AI in the diagnosis of OC. Special consideration was given to the diagnostic accuracy of AI and its ability to identify the early stages of OC.

**Materials and methods:**

From the date of inception to December 2021, 4 databases (PubMed, Scopus, EBSCO, and OVID) were searched. Three independent authors selected studies on the basis of strict inclusion criteria. The risk of bias and applicability were assessed using the prediction model risk of bias assessment tool. Of the 606 initial records, 17 studies with a total of 7245 patients and 69,425 images were included. Ten statistical methods were used to assess AI performance in the included studies. Six studies used supervised machine learning, whilst 11 used deep learning. The results of deep learning ranged with an accuracy of 81% to 99.7%, sensitivity 79% to 98.75%, specificity 82% to 100%, and area under the curve (AUC) 79% to 99.5%.

**Results:**

Results obtained from supervised machine learning demonstrated an accuracy ranging from 43.5% to 100%, sensitivity of 94% to 100%, specificity 16% to 100%, and AUC of 93%.

**Conclusions:**

There is no clear consensus regarding the best AI method for OC detection. AI is a valuable diagnostic tool that represents a large evolutionary leap in the detection of OC in its early stages. Based on the evidence, deep learning, such as a deep convolutional neural network, is more accurate in the early detection of OC compared to supervised machine learning.

## Introduction

According to the Global Cancer Statistics of 2018, oral cancer (OC) (International Classification of Disease [ICD]: 10 C00–06) is the 11th most frequently reported cancer worldwide, with over 640,000 new cases reported annually.^1^ Despite major improvements in cancer diagnosis and treatment modalities, morbidity and mortality rates of OCs remain high, particularly in advanced stages (T3 and T4).^2^, ^3^, ^4^, ^5^ Although histologic evaluation of biopsies by an oral pathologist remains the gold standard for diagnosing OC, it is liable to subjective judgment due to discrepancies in interpretation and variability of results.^6^ Therefore, alternative methods that are anticipated to provide more accurate, fast, and standardised diagnosis and improve OC patient survival rates are needed.

Artificial intelligence (AI) is an area of computer science that can be defined as a machine's capacity to emulate a human's cognitive capacity. The term “artificial intelligence” refers to a wide range of methodologies. For instance, deep learning is a potentially revolutionary technology that attempts to model high-level abstractions in medical imagery to derive diagnostic meanings.

It is vital to remember that AI is a broad term that encompasses 2 distinct branches: traditional machine learning and deep learning. Traditional machine learning uses algorithms and computer processes to calculate information and recognise patterns from input data and then offers a quantified judgment as a diagnostic result regarding the nature and behaviour of the lesion.^3^ Traditional machine-learning approaches are further divided into supervised and unsupervised methods. The supervised technique relies on the machine learning model being trained to validate the inputs and outputs that are used as the model's ground truth against which the diagnostic input is tested.^7^ In contrast, the unsupervised techniques are machine learning models that are not built upon preordained values; hence, it uses extraction and mining methods to explore common hidden features from the input data or specimen.^8^ Deep learning or neural networks, which are regarded as a subset of machine learning, are computational techniques based on the formation of nonlinear processing units with multiple hidden layers to learn and comprehend input and associate it with the output. Unlike classical machine learning, deep learning can process large-scale data, given the intricacy and abstraction of data, and explore complex relations between the input and output.^9^^,^^10^

Recently, there has been a significant surge in research on AI-based technologies for medical imaging and diagnosis.^11^ The reason for implementing AI in the field of oncology is its potential to improve the accuracy and efficacy of cancer screening.^6^ AI technologies are effective in identifying breast, lung, and oral cancers.^12^, ^13^, ^14^ These techniques are currently being evaluated for inclusion in diagnostic systems, particularly for disease screening in resource-constrained situations, where trained doctors and experts are in short supply.^15^, ^16^, ^17^

Because AI has always been under constant investigation and development, many reviews have been conducted during the last decade. However, there is a lack of emphasis on the accuracy or sensitivity of the method in the early detection of OC.

The use of AI can reduce the effort required for sceening and analysis of large data sets during detection of malignant lesions.^6^ However, more research on the use of AI in the diagnosis of OC is required. Primarily, the accuracy and efficiency of AI in recognizing OC in comparison to a trained clinician must be evaluated, along with detection at an early stage.

This systematic review was conducted to critically evaluate the available evidence concerning the accuracy and efficiency of utilizing AI in diagnosing OC and whether AI can detect OC lesions in their early stages as precisely as a clinician can.

## Methodology

### Protocol

This systematic review adhered to the Preferred Reporting Items for Systematic Reviews and Meta-Analyses (PRISMA) statement for reporting systematic reviews.^18^ The systematic review protocol was registered on the PROSPERO platform (CRD42021288107).

### Focused question

Is AI effective in providing an accurate diagnosis for the early detection of OC?

The question for the current systematic review was adopted to follow the PICO criteria:

P: Oral squamous cell carcinoma (OSCC) cases

I: AI (machine and deep learning)

C: Cancerous vs noncancerous images

O: Accuracy of AI in the early detection of OC

#### Literature search

From inception to November 30, 2021, the University of Sharjah Library was used to conduct the search, which included access to 4 databases: PubMed, Scopus, EBSCO, and OVID. The publications collected were published between 2000 and 2021, ensuring that the literature gathered provided a comprehensive picture of AI advancement in the field of OC detection and diagnosis. A set of keyword combinations “oral cancer” [MeSH term] AND “machine learning” [MeSH term] OR “deep learning” [MeSH term] OR “neural network” [MeSH term]) was used to search the literature in all 4 databases to ensure that all relevant articles were screened.

A manual search of the following dental journals was also performed: *Journal of Oncology, Journal of Oral Diseases, Journal of Oral Pathology & Medicine and Oral Surgery Oral Medicine, Oral Pathology Oral Radiology, International Journal of Oral and Maxillofacial Surgery, European Journal of Craniomaxillofacial Surgery, British Journal of Oral and Maxillofacial Surgery*, and *Journal of Craniofacial Surgery*.

Additional research was conducted on the basis of the reference lists of the discovered studies and pertinent reviews on the issue. Furthermore, ClinicalTrials.gov, www.centerwatch.com/clinical trials, and www.clinicalconnection.com were used to search the web databases for information on ongoing clinical studies.

### Inclusion and exclusion criteria

The inclusion criteria were as follows:

1. Human experimental or observational studies that have employed AI technology to identify OCs.
2. Research comparing physicians’ diagnostic outcomes against AI for OC.
3. The samples collected should be in the form of histologic or photographic images.
4. Full-text, English-language studies that reported accuracy, sensitivity, specificity, and/or area under the curve (AUC).

The exclusion criteria were as follows:

1. Studies with fewer than 10 patients.
2. Studies including individuals with recurrent OC.
3. Animal studies.
4. Literature reviews, case reports, short communication, non-English studies, personal viewpoints, letters to editors, and conference abstracts.

### Study selection and data extraction

The titles, abstracts, and full texts of the relevant studies were examined separately by 3 reviewers, and any disagreements were resolved by consensus. The reviewers retrieved the required information from eligible studies. The following data were collected for each study (when available): author, year, country, sample type, sample size, learning machine and training set/cycle, statistical findings (accuracy, sensitivity, specificity, and AUC), and the main outcomes (Table 1).

**Table 1:** Characteristics of the included studies.

*No.*	*Author, year, country*	*Sample number*	*Sample type*	*Learning machine/training cycle and sets*	*Statistical findings (AUC, sensitivity, specificity, etc)*	*Main outcome*
1	Welikala et al^7^ India	No. of patients = 1085 No. of images = 2155 Training images = 1744 images Validation images = 207	Photographic images	1. Image classification: ResNet-101 neural network 2. Object detection: Region proposal network (RPN) and detection network	Image classification: -***Images that contained lesion:****P* = 84.77%, *R* = 89.51%, F1 87.07% -***Object detection***: *P* = 46.61%, *R* = 37.16%, F1 = 41.35%	Initial results demonstrate the effectiveness of deep learning and are encouraging when we consider the scale of the problem.
2	Majumder et al^8^ India	No. of patients = 114 HG-OSCC = 45 patients with 225 tissue sites LG-OSCC = 23 patients with 83 tissue sites Leukoplakia = 6 patients with 40 tissue sites Normal = 30 patients with 225 tissue sites	Oral tissue biopsies	Total principal component analysis regression (TPCR), based direct multiclass discrimination algorithm. Training cycle and set = 4 training sets and 4 validation sets	TPCR accuracy with 4 classes ***-Training Data:*** HG-OSCC = 94%, LG-OSCC = 100%, leukoplakia = 100%, normal = 100% -***Cross-validation data:*** HG-SCC = 90%, LG-SCC = 90%, leukoplakia = 85%, normal = 88%	TPCR was found to provide satisfactory performance in classifying the tissue sites in 4 different low classes: high-grade squamous cell carcinoma, low -grade squamous cell carcinoma, leukoplakia, and normal squamous tissue.
3	Das et al^20^ India	No. of patients = 43 Total No. of images = 126 with 3 images from each slide; (normal = 2, LG-OSCC = 25, HG-OSCC = 15)	Histologic slide image	DCNN **Training cycle** and set = 20 epochs	**Epithelia segmentation:** AC = 98.42%, SN = 97.76% **Keratin pearls detection:** AC = 96.88%	The proposed CNN has higher accuracy results and better performance in the segmentation of tissue layer and keratin pearl detection of the histologic image of OSCC than the existing state of the art for epithelial layer segmentation.
4	Uthoff et al^21^ India	Number of patients = 190 Number of images = 170 image pairs Normal class = 86; suspected OSCC = 84	Autofluorescence image and white light image	CNN Training cycle and set = 80 epochs	**On-site specialist**: AUC = 0.908, SN = 0.8500, SP = 0.8875, PPV = 0.8767, NPV = 0.8549 **Remote specialist**: SN = 0.9259, SP = 0.8667, PPV = 0.9494, NPV =0.8125	With suspect areas outlined, the combination of WLI and AFI provides the most information about the type of lesion and the size of the affected area. Compared to on-site specialists, the remote specialist was able to diagnose patients correctly with the help of the proposed device with high value and performance.
5	Song et al^22^ India	No. of patients = 12 No. of images = 35 images	P53 immunostained tissue section	Supporting vector machine **Training cycle and set** = not mentioned	Blue component: AC = 98.01%, SN = 98.86%, SP = 94.74%	The experimental result, blue component of automatic technique, has performed well in classification as well as detecting immunopositivity of tissue images. Also, they found that the immunopositive ratio values of both manual and automatic techniques were equal.
6	Song et al^23^ India	2350 cheek mucosa images	The intraoral data set of cheek mucosa images	Learning machine: Bayesian deep network training = 300 epochs	AC = 90%	The performance can be further improved by referring more patients. The experiments show that the model is capable of identifying difficult cases needing further inspection.
7	Jeyaraj et al^24^ India	Total image in BioGPS data = 100 (tumor = 65, normal = 35) Total images in TCIA archive = 500 (tumor = 450, normal = 50) Total image in GDC data set = 700 (tumor = 625, normal = 75)	Multidimensional hyperspectral image	Partitioned DCNN **Training cycle** and set = not mentioned	***DCNN algorithm*** (with 100-image set): AC = 91.4%, SP = 91%, SN = 94%, AUC = 0.94) ***Proposed partitioned CNN algorithm*** (with 500-image set): AC = 94.5%, SP = 98%, SN = 94%, AUC = 0.965)	Proposed partitioned CNN had higher accuracy results compared with the other classifier SVM and DBN, and the accuracy increased by 4.5% when a large number of cancer patient data sets were used in the training phase.
8	Rahman et al^25^ India	Total No. of slides = 42 Normal = 13, (OSCC lesion = 29) Total No. of images of nuclei acquired from slide = 720 (normal = 237, malignant = 483)	Histopathologic slide	1. Tree-based classification 2. Logistic regression 3. K-nearest neighbour classifier 4. SVM classifier 5-Linear discriminant analysis **Training cycle and set** = Cycles: 5, training sets: 4, testing sets: 1	**For texture, shape, and colour features:** 1. SN = 99.2%, SP = 99.8%, AC = 99.4% 2. SN = 100%, SP = 100%; AC = 100%; 3. SN = 99.2%, SP = 16.1%, AC = 43.5% 4. SN = 100%, SP = 100%, AC = 100% 5. SN = 99.6%, SP = 100%, AC = 99.9%	Accurate results for colour, shape, and texture features using the classification were achieved. The in-depth analysis showed that SVM and linear discriminant classifiers gave the best results for texture and colour features.
9	Shahul Hameed et al^26^ India	No. of patients = 40 -27 slides -118 normal cells -334 malignant slides -Total of 452 extracted morphologic features	Histologic images	1. Decision tree classifier 2. SVM 3. K-nearest neighbour 4. Discriminant analysis 5. Logistic regression **Training cycle and set** = not mentioned	Accuracy of: -Decision tree = 99.78% -Linear discriminant = 93.6% -Logistic regression = 62.9% -SVM = 93.6% -K-nearest neighbour = 54.3%	The decision tree yielded the highest accuracy.
10	Duran-Sierra et al^27^ USA	57 patients for tissue biopsy examination of suspicious oral epithelial precancerous or cancerous lesions	Multispectral auto-fluorescence lifetime imaging	**Learning machine:** 1. Linear discriminant analysis, quadratic	SN = 94% SP = 74% F1 score = 0.85	The model using spectral-only features was SVM. LOGREG was the best performing classification, WhileQDA was the best-performing model using time-resolved-only features.
11	Schwarz et al^28^ USA	Patient No. with oral lesion = 60, with 154 sites -Normal volunteers = 64, with 270 sites	Spectroscopy probe, biopsy	SVM: linear discriminant analysis Training cycle and set = not mentioned	SN = 82%, SP = 87%, AUC = 0.93	Differences in oral spectra were observed in (1) neoplastic vs non- neoplastic sites, (2) keratinised vs nonkeratinised tissue, and (3) shallow vs deep depths within oral tissue. Algorithms based on spectra from 310 nonkeratinised anatomic sites (buccal, tongue, floor of mouth, and lip) yielded an area under the receiver operating characteristic curve of 0.96 in the training set and 0.93 in the validation set.
12	Song et al^29^ USA	6211 pairs of intraoral images from 5025 patients	Intraoral images	**Learning machine =** dual-modality mobile-based classification using deep learning model MobileNet/**Training =** 300 epochs.	AC = 81%, SN = 79%, SP = 82%	The proposed method achieved 81% accuracy for distinguishing normal/benign lesions from clinically suspicious lesions.
13	Fu et al^30^ China	No. of images: -Initial data set = 44,409 images -Algorithm development = 5575 -IVD = 401 -Secondary analysis = 170 -EVD = 420 photographs -CVD = 666 photographs	Photographic images	**Learning machine:** DCNN **Training cycle and set** = not mentioned	**IVD:** AUC = 0.983 (95%), SN = 94.9%, SP = 88.7%, AC = 91.5% -**Secondary analysis on IVD:** AUC = 0.995, SN = 97.4%, SP = 93.5%, AC = 95.3% **EVD:** AUC = 0.935, SN = 89.6%, SP = 80.6%, AC = 84.1% **CVD:** AUC = 0.97, SN = 91.0%, SP = 93.5%, AC = 92.3% Overall accuracy = 92.3%	This deep neural network is helpful in identifying these very small OSCC lesions in high-risk individuals, achieving a promising result (AUC = 0.995) during the secondary analysis on internal validation data set, which is comparable to a human specialist.
14	Lin et al^31^ China	Oral lesion images = 688 Normal mucosa images = 760	Photographic images	**Learning machine =** smartphone-based image diagnosis with deep learning network HRNet/**Training** = 15, 30, and 45 epochs.	SN = 83%, SP = 96.6%, *P* = 84.3%, F1 = 83.6%	The performance of HRNet model achieved slightly better performance when compared to VGG16, ResNet50, DenseNet169. Also the F1 score was higher by 8% when a centre positioning method was used.
15	Aubreville et al^32^ Germany	No. of patients = 12 Total No. of images = 7894 (Normal alveolar ridge = 1951, normal inner labium = 1317, normal hard palate = 811, and OSCC lesion = 3815)	Confocal laser endomicroscopy images	Learning machine: DCNN **Training cycle and set** = 60 epochs	**Proposed CNN:** AC = 88.3%, SN = 86.6%, SP = 90.0%, AUC = 0.96	Present CNN approach using ppf method significantly outperforms conventional approach, that is, textural feature-based machine for CLE image recognition.
16	Warin et al^33^ Thailand	700 clinical oral photographs	Oral photographs.	Learning machine: DenseNet121 and Faster R-CNN network. **Training**: not mentioned	**DenseNet121: *P*** = 100%, ***R*** = 99%, F1 = 99%, SN = 98.75%, SP = 100%, AUC = 0.99 **Faster R-CNN:** *P* = 76.67%, *R* = 82.14% F1 = 79.31%, AUC = 0.79	The DenseNet121 and faster R-CNN algorithm were proved to offer the acceptable potential for the classification and detection of cancerous lesions in oral photographic images.
17	Jubair et al^34^ Jordan	Total patients = 543 Total images: 716 Suspicious images (OC and oral dysplasia) = 236 Benign lesions = 480	Photographic images: tongue	**Learning machine:** CNN (EfficientNet-B0) **Training:** 5 epochs, Bootstrapping = 120 repetitions	SP = 84.5%, SN = 86.7%, AC = 85.0%, AUC = 0.911	Deep CNN using EfficientNet-B0 transfer model can be used for detection of cancerous or potentially malignant oral lesions with high levels of accuracy, sensitivity, and specificity.

AC, accuracy; AFI, auto-fluorescence imaging ; AUC, area under the curve CLE, confocal laser endomicroscopy; CNN, convolutional neural network; CVD, clinical validation dataset; DBN, deep belief network; DCNNdeep convolutional neural network; EVD, external validation dataset; GDC, genomic data commons; GPS, BioGPS data portal; HG-SCC, high grade squamous cell carcinoma; ; IVD, internal validation dataset; ; LG-OSCC, low grade squamous cell carcinoma; ; OSCC, oral squamous cell carcinoma; NPV, negative predictive value;*P,* precision; ppf, patch probability fusion; PPV, positive predictive value; QDA, quadratic discriminant analysis; SN, sensitivity; SP, specificity; TCIA, the cancer imaging archive; WLI, white light imaging; SVM, support vector machine; OC, oral cancer.

### Risk of bias and quality of the studies assessment

A prediction model risk of bias assessment tool (PROBAST tool) for nonrandomised studies was used to assess the risk of bias and applicability of the studies^19^ (Table 2). PROBAST is a collection of 20 questions from 4 different domains (participants, predictors, outcomes, and analysis). Yes, probably yes, probably no, no, or no information was provided as response for each question. A domain should have had all questions answered with yes or probably yes to be considered low risk. If at least one question in a domain was answered no or probably no, the study was classified as having a high risk of bias unless the assessors determined that the risk was low or uncertain based on the overall indicators. Similarly, to be considered an unclear risk, at least one domain was rated as having an unclear risk of bias, whereas the other domains were rated as having a low risk of bias.

**Table 2:** PROBAST tool to assess the risk of bias and applicability.

*Author*	*Type of study*	*Risk of bias*				*Applicability*			*Overall*	
		*Participant selection*	*Predictors*	*Outcome*	*Analysis*	*Participant selection*	*Predictors*	*Outcome*	*Risk of bias*	*Applicability*
Welikala et al^7^ India	Development and validation	**–**	**+**	**+**	**+**	**–**	**+**	**+**	**–**	**–**
Majumder et al^8^ India	Development and validation	**–**	**+**	**–**	**–**	**–**	**+**	**+**	**–**	**–**
Das et al^20^ India	Development and validation	**+**	**+**	**+**	**+**	**+**	**+**	**+**	**+**	**+**
Uthoff et al^21^ India	Development and validation	**+**	**+**	**+**	**+**	**+**	**+**	**+**	**+**	**+**
Song et al^22^ India	Development	**+**	**+**	**+**	**+**	**+**	**+**	**+**	**+**	**+**
Song et al^23^ India	Validation	**+**	**+**	**+**	**+**	**+**	**+**	**+**	**+**	**+**
Jeyaraj et al^24^ India	Development and validation	**+**	**+**	**+**	**+**	**+**	**+**	**+**	**+**	**+**
Rahman et al^25^ India	Development	**+**	**+**	**+**	**+**	**+**	**+**	**+**	**+**	**+**
Shahul Hameed et al^26^ India	Development and validation	**+**	**+**	**+**	**+**	**+**	**+**	**+**	**+**	**+**
Duran-Sierra et al^27^ USA	Validation	**–**	**+**	**?**	**+**	**-**	**-**	**+**	**–**	**–**
Schwarz et al^28^ USA	Development and validation	**+**	**+**	**+**	**+**	**+**	**+**	**+**	**+**	**+**
Song et al^29^ USA	Development and validation	**+**	**+**	**+**	**+**	**+**	**+**	**+**	**+**	**+**
Fu et al^30^ China	Development and validation	+	+	–	–	+	+	–	–	+
Lin et al^31^ China	Development	**+**	**+**	**+**	**+**	**+**	**+**	**+**	**+**	**+**
Aubreville et al^32^ Germany	Development and validation	**+**	**+**	**+**	**+**	**+**	**+**	**+**	**+**	**+**
Warin et al^33^ Thailand	Development and validation	**+**	**+**	**+**	**+**	**+**	**+**	**+**	**+**	**+**
Jubair et al^34^ Jordan	Development and validation	**+**	**+**	**?**	**+**	**+**	**+**	**+**	**+**	**+**

+, low risk of bias/low concerns regarding applicability; −, high risk of bias/high concerns regarding applicability; ?, unclear risk of bias/unclear concerns regarding applicability.

### Data synthesis

The collected data and main findings are presented in the form of narrative synthesis. Due to the heterogeneity amongst the selected studies, formal quantitative syntheses were not conducted.

## Results

### Literature search

**Fig. 1:**
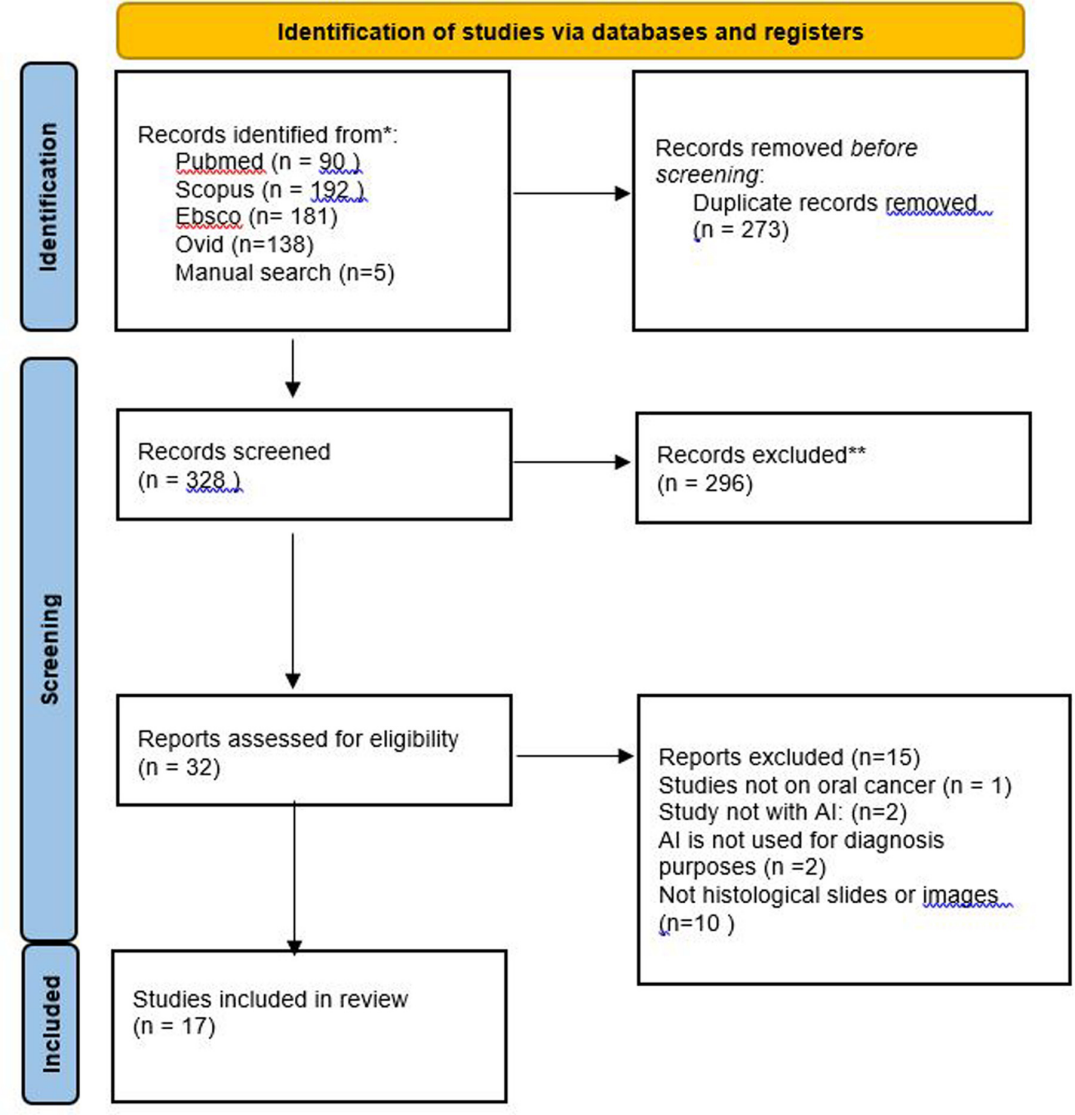
PRISMA flowchart of the studied sample.

The kappa value was 0.85; therefore, an agreement amongst the 3 investigators was almost perfect. Through electronic and manual searches, 606 articles were identified (PubMed, 90; Scopus, 192; EBSCO, 181; OVID, 138; and manual search, 5) (Figure 1). After the duplicate removal process, 328 articles remained. The titles and abstracts of the 328 records were examined on the basis of predefined eligibility criteria. Consequently, 296 articles were excluded because they were off-topic. The full text of the remaining 32 articles was carefully read by 2 reviewers for potential inclusion. The articles were narrowed down to 17 articles selected to draw the results of the systematic review. However, the remaining 15 articles were excluded because either their AI model was utilised for reasons other than OC diagnosis, AI was not utilised for OC early detection purposes, or samples used were not presented as histologic or photographic images. The process of study selection is documented in the PRISMA flowchart in Figure 1.

### Study quality assessment

Using the PROBAST checklist, 13 studies were assessed as having a low risk of bias, and 4 studies were rated as having a high risk of bias. In terms of applicability, 14 studies were reasonably applicable (Table 2).

### Study characteristics

#### Demographic characteristics

The total number of patients from the included studies was 7245, and the total number of images analysed was 69,425. Seventeen studies were from various countries, with India accounting for 9 of them.^7^^,^^8^^,^^20^, ^21^, ^22^, ^23^, ^24^, ^25^, ^26^ Three studies^27^, ^28^, ^29^ were conducted in the United States, 2^30^^,^^31^ were performed in China, and the other studies were carried out in Germany,^32^ Taiwan,^33^ and Jordan.^34^

The sample size was calculated on the basis of the number of patients recruited, and 4 studies^8^^,^^21^^,^^25^^,^^27^ had fewer than 100 patients. The smallest number of patients was 12.^18^ The largest sample size was 502,529. In terms of image count, the minimum number of histologic images was 3522, whilst the largest was 44,40,930.

### Study designs

All the selected studies were clinical trials. Nine were case-control studies,^7^^,^^8^^,^^20^^,^^21^^,^^23^^,^^28^^,^^30^^,^^33^^,^^34^ 7 were comparative studies,^22^^,^^24^, ^25^, ^26^, ^27^^,^^29^^,^^31^ and only one was a retrospective study,^30^ with several of them employing various statistical procedures for a range of AI technologies.

**Fig. 2:**
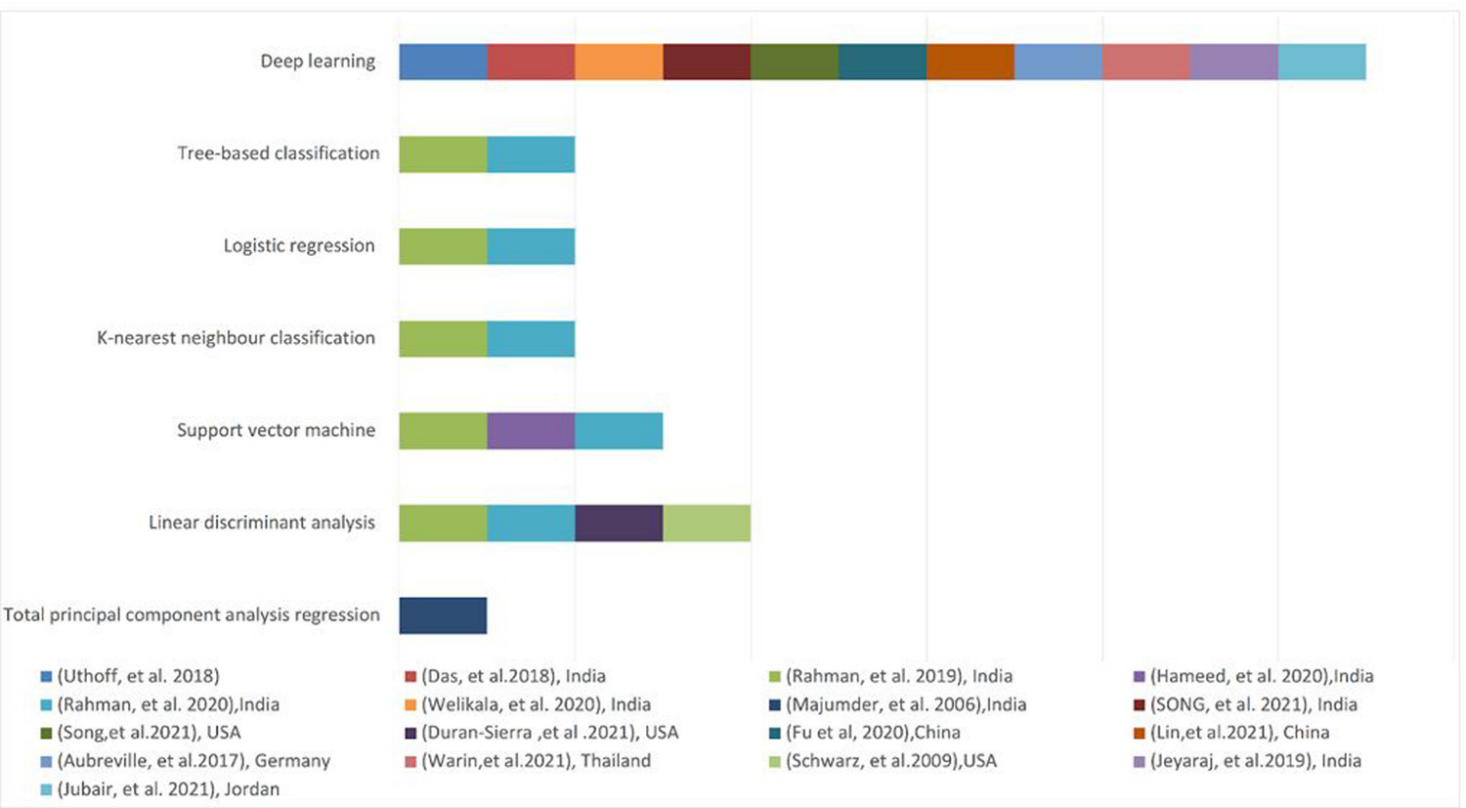
Types of artificial intelligence (AI) used by each study for the purpose of oral cancer diagnosis, with 11 studies utilised deep learning and 6 studies used supervised machine learning.

The studies provide 7 forms of AI, including several types of supervised classical machine learning models and deep learning. In most investigations, deep learning has been used to detect OCs. Nonetheless, in terms of frequency of use, deep learning (convolutional neural network [CNN]) was used in 11 studies,^7^^,^^20^^,^^21^^,^^23^^,^^24^^,^^29^, ^30^, ^31^, ^32^, ^33^, ^34^ whilst 6 studies used machine learning.^8^^,^^22^^,^^25^, ^26^, ^27^, ^28^ The most frequently used subtype of the supervised machine learning approach is the support vector machine, which was used in 4 studies.^25^, ^26^, ^27^, ^28^ Three studies used smartphone applications,^14^^,^^21^^,^^31^ all of which used deep learning techniques. Figure 2 compares the AI models used along with their frequencies amongst the 17 studies.

### Study comparator

Uthoff et al sorted samples into the suspicious and nonsuspicious categories.^21^ Other studies^8^^,^^20^^,^^23^^,^^24^^,^^27^, ^28^, ^29^^,^^31^ offered an AI model that could categorise lesions as normal, precancerous, or cancerous, with or without additional categorisation of the samples into various stages of OC. Five studies^25^^,^^26^^,^^30^^,^^32^^,^^33^ presented AI methods to categorise samples using binary classification as normal or malignant. Jubair et al^34^ divided the samples into benign or suspicious (malignant or premalignant). Furthermore, Schwarz et al presented an AI that can categorise samples into a range of normal to mild dysplasia (negative) vs moderate dysplasia to cancer (positive).^28^

Welikala et al divided the samples into 5 categories: no lesion, no referral needed, refer for other reasons, refer- low risk of potentially malignant disorders (OPMD), and refer cancer high-risk OPMD.^7^ Other studies^22^^,^^27^ categorised samples as positive or negative based on staining intensity.

### Study outcome

Table 1 summarises the findings of the study. Various statistical tests have been used to test and verify the efficacy of machine learning in OC diagnosis. Accuracy, sensitivity, specificity, and AUC were employed in most of the investigations. Eleven studies utilised accuracy to assess the efficacy of AI technology.

The overall accuracy rate ranged from 43.5%^25^ to 100%.^8^ Eight of the 11 articles had an accuracy of at least 90%.^8^^,^^20^^,^^22^, ^23^, ^24^, ^25^, ^26^^,^^30^ Three investigations had an accuracy rating of less than 90%.^29^^,^^32^^,^^34^ Deep learning yielded an accuracy range between 81%^29^ and 96.88%.^20^ However, the range of values for supervised machine learning ranges from 43.5%^25^ to 100%.^8^

Thirteen studies examined the effectiveness of AI in diagnosing OC in terms of its sensitivity. Seven studies^20^^,^^22^^,^^24^^,^^25^^,^^27^^,^^30^^,^^33^ reported a sensitivity of 90% or more. Moreover, 6 studies^21^^,^^28^^,^^29^^,^^31^^,^^32^^,^^34^ reported a sensitivity of less than 90%. The sensitivity of deep learning ranged from 79%^29^ to 98.75%.^33^ However, supervised machine learning ranged between 94%^27^ and 100%.^25^

Specificity was assessed in 12 studies to measure AI efficiency. Six studies had a result value equal to or greater than 90%.^22^^,^^24^^,^^25^^,^^31^, ^32^, ^33^ In contrast, six investigations reported a specificity result value of less than 90%.^21^^,^^27^, ^28^, ^29^, ^30^^,^^34^

For deep learning, specificity ranged between 80.6%^30^ and 100%,^33^ whereas supervised machine learning scored between 16% and 100%.^25^

Seven of the 17 studies employed AUC to assess the efficiency of the AI machine. AUC values of more than 0.9 were found in 7 investigations.^21^^,^^24^^,^^28^^,^^30^^,^^32^, ^33^, ^34^

Some studies utilised different statistical methods to assess AI performance, such as the F1 score,^7^^,^^27^^,^^31^^,^^33^ recall,^7^^,^^33^ precision,^7^^,^^31^^,^^33^ positive predictive value, and negative predictive value.^21^

## Discussion

The main goal of this systematic review was to evaluate the effectiveness of AI in detecting and screening for OC using photographic and histologic images. Most of the studies included in this systematic review showed that machine learning models can detect OC with excellent accuracy, sensitivity, and specificity. Current advancements in machine learning algorithms allow the detection of OC using an efficient and noninvasive technique with a performance comparable to that of human specialists.^30^ Although the oral cavity is accessible during a normal checkup, many cancers are not discovered until they are advanced.^7^ Experts can detect OCs through visual inspection based on the clinical appearance of the lesion. Using AI as a more accurate and quick method for diagnosing OC in its early stages may be one of the most effective ways to decrease death rates. Currently, there is growing interest in using AI in oncology to improve the accuracy and efficacy of screening suspected lesions.

### Machine learning vs deep learning methods

All selected studies in this systematic review utilised supervised machine learning and deep learning models, with 6 studies using supervised machine learning and 11 studies using deep learning methods (Figure 2). Studies that used deep learning had an accuracy range of 72% to 99.2%, whereas machine learning had a range of 43.5% to 100%.^7^^,^^8^^,^^20^, ^21^, ^22^, ^23^, ^24^, ^25^, ^26^, ^27^, ^28^, ^29^, ^30^, ^31^, ^32^, ^33^, ^34^ Modalities employing deep learning show consistent results with a narrow range of accuracy, whereas machine learning shows a wide range of differences, making the machine learning results or performance somewhat unpredictable.

### Overall performance

Regarding the overall performance of deep learning, the highest result was reported in 4 studies. In a study by Uthoff et al, who used a deep learning approach using smartphone data transmission power to discriminate between suspicious and nonsuspicious lesions, they obtained a minimum risk of bias based on the probability scoring system with an AUC of 0.908.^21^ In contrast, the Gabor texture descriptor was employed by Das et al to identify keratin pearl from non-pearl regions.^20^ They discovered that the colours of the 3 primary constituent layers, epithelium, subepithelial, and keratin areas, could be discriminated.^20^ Fu et al analysed 44,409 images, and they yielded a high accuracy even though a large sample was utilised.^30^ Fu et al employed a detection network to take an oral photograph as the input and create a single bounding box that indicates the probable lesion. The lesion region was trimmed as a candidate patch based on the detection results obtained in the first step. The candidate patch was then provided to a classification network, which produced a list of 2 confidence ratings in the range of 0 to 1 for patients with OSCC and controls.^30^ Because the photographs used to train the deep neural networks may not accurately reflect the diversity and heterogeneity of oral disease lesions, the algorithm cannot make reliable predictions for other oral lesions. Seven studies used the AUC to evaluate the proposed machine learning method. The highest AUC score was 99.5% for the deep CNN using photographic images in the secondary analysis of the internal validation data set.^30^ Rahman et al scored the highest value in terms of accuracy, sensitivity, and specificity using a support vector machine classifier and logistic regression.^25^ In contrast, the K-nearest neighbour classifier scored the lowest for accuracy, specificity, and AUC.^33^

### AI accuracy for histopathologic images

The histopathologic analysis is the gold standard for the detection and diagnosis of OC. However, this method relies on subjective analyses, which makes screening accuracy by the clinician subjective.^6^ When histopathologic samples are examined for OC, certain features and characteristics allow the pathologist to determine whether a patient presents with malignancy and to identify the stage. Sometimes, as the manual evaluation of samples for diagnostic features requires quantification, there is a chance for error, which inevitably leads to inaccurate results.^6^ Consequently, AI has reduced such errors and improved the efficiency and accuracy of detecting the cytologic and histologic features of OC. Moreover, AI technology can process large sample sizes to detect OC. Two types of samples were used in the selected studies: biopsy and histologic samples and photographic images. Six studies used biopsy and histologic samples.^8^^,^^20^^,^^22^^,^^25^, ^26^, ^27^ Some studies that examined cellular changes to differentiate malignant samples from normal and abnormal cell nuclei have defined them as a marker.^22^^,^^25^^,^^26^ Das et al inspected epithelial changes by detecting keratin pearls in the oral mucosa of patients with OC using the proposed segmentation method.^20^ They quantified the keratinisation layer, which was successful with their proposed CNN machine because this parameter is significant in determining the stage of OC.^20^

### Future perspectives, translational value, and limitations

Researchers have found that deep learning aids pathologists in the effective multiclass classification of cancer. This enables the oncology team to deliver an effective treatment plan, whilst minimizing the overall workload. Additionally, deep learning models can categorise patients into high- or low-risk categories, thus aiding oncologists in deciding whether to choose a radical or conservative treatment approach for the patient. This could exclude patients in low-risk categories from the harmful effects of the radical approach.^35^^,^^36^ Although these factors strongly favour the translation of AI-based research into clinical oncology practice, there are a few limitations. Privacy and confidentiality of patient data remain major hurdles in the clinical application of AI in oncology.^37^ There is also a question of owning the responsibility (doctor or software) in case of an error in AI-based analysis. Apart from these factors, the patient's autonomy and relationship with the treating clinician are affected by the introduction of AI in oncology practise.^37^

## Conclusions

This systematic review supports that machine learning yields accurate results for detecting OC, which is of great assistance for pathologists to improve their diagnostic results and minimise the chance of error. Furthermore, studies that ranked the strongest based on their evidence have applied deep learning (neural networks), which indicates a high performance and thus is more accurate.

## Author contributions

**Al-Rawi NH:** Conceptualisation (lead); supervision (lead); data curation (equal); formal analysis (equal); investigation (equal); methodology (equal); writing original draft (equal).

**Sultan A:** Validation (equal); data curation (equal); formal analysis (equal); investigation (equal); methodology (equal); writing original draft (equal).

**Rajai B:** Validation (equal); data curation (equal); formal analysis (equal); investigation (equal); methodology (equal); writing original draft (equal).

**Shuaeeb H:** Validation (equal); data curation (equal); formal analysis (equal); investigation (equal); methodology (equal); writing original draft (equal).

**Alnajjar M:** Validation (equal); data curation (equal); formal analysis (equal); investigation (equal); methodology (equal); writing original draft (equal).

**Alketbi M:** Validation (equal); data curation (equal); formal analysis (equal); investigation (equal); methodology (equal); writing original draft (equal).

**Mohammad Y:** Validation (equal); data curation (equal); formal analysis (equal); investigation (equal); methodology (equal); writing original draft (equal).

**Shetty SR:** Validation (lead); data curation (equal); formal analysis (equal); investigation (equal); methodology (equal); writing original draft (equal).

**Mashrah MA:** Conceptualisation (lead); data curation (equal); formal analysis (equal); investigation (equal); methodology (equal); writing original draft (equal).

## Conflict of interest

None disclosed.
